# No travel worsens depression: reciprocal relationship between travel and depression among older adults

**DOI:** 10.1186/s12991-022-00405-2

**Published:** 2022-08-10

**Authors:** Seungjae Hyun, Yeonjin Lee, Sangshin Park

**Affiliations:** 1 Graduate School of Urban Public Health & Department of Urban Big Data Convergence, University of Seoul, Seoul, Republic of Korea; 2 Department of Sociology, Kookmin University, 77 Jeongreung-ro, Seongbuk-gu, Seoul, Republic of Korea

**Keywords:** Travel, Depression, Korea, Korean Longitudinal Study of Ageing

## Abstract

**Background:**

The aim of this study was to examine the bidirectional relationship between depression and travel.

**Method:**

We analyzed 8524 participants’ data obtained from the 2008 to 2016 waves of the Korean Longitudinal Study of Ageing, a prospective cohort study. Depression was diagnosed using the Center for Epidemiological Studies Depression Scale: 10-Items (CES-D10), with scores of 4 or higher indicating depression. We used a generalized estimating equation and a cross-lagged panel model for statistical analysis.

**Results:**

Participants who had not traveled for one year had a 71% higher risk of suffering from depression in the following year than did those who had traveled [relative risk (RR) = 1.71, *P* < 0.001], and participants with depression had more than double the increased risk of not traveling than did those not currently suffering from depression (RR = 2.08, *P* < 0.001). The cross-lagged panel model confirmed the vicious cycle involving the amount of travel and score on the CES-D10; individuals who traveled more frequently were more likely to have lower scores on the CES-D10 (coefficient = − 0.04 to − 0.03, *P*s < 0.01), and individuals with higher scores were less likely to travel (coefficient = − 0.06 to − 0.03, *P*s < 0.01).

**Conclusions:**

The risk of depression increases for people who do not travel, and a reciprocal relationship exists between travel and depression.

## Introduction

Depression is characterized as an emotion-regulation disorder caused by a failure to modulate negative emotions in behavioral, cognitive, and physiological processes [1]. Depression has become a major public health issue throughout the world, particularly contributing to the burden of disease among older adults. Its prevalence rate tends to peak in people 55–74 years of age (above 7.5% in females and above 5.5% in males) [2]. Depression is associated with many diseases such as diabetes mellitus [3], stroke [4], rheumatoid arthritis [5], frailty [6], cancer [7], cardiovascular disease, and all-cause mortality [8]. According to a World Health Organization report, the number of people living with depression was 322 million in 2015, the prevalence of depression was about 4.4%, and the rate of diagnosable depressive symptoms increased by 18.4% between 2005 and 2015 [9]. One strategy for overcoming depression disorder is distraction, which involves intentional diversion of attention from negative stimuli [10, 11]. Travel means to go from one place to another for leisure, which helps people escape their daily living environment (DLE) DLE and makes for pleasant diversion, encouraging leisure time and relaxation [12].

According to the activity theory older adults tend to have higher intentions to engage in informal social activities compared to younger counterparts [13]. Laferrère [14] finds that older adults who exit labor force are more likely to participate in social gatherings, clubs, and non-paid activities. Socio-emotional selectivity theory also suggested that older adults prioritize emotional needs and prefer to participate in an informal gathering, maintaining peripheral networks that give positive stimulus [15]. To continue having motivations to find positive stimulus such as to go on travel while growing older can be a good way to improve mental health. Taking a trip gives plausible chances to engage in informal gatherings, make minor networks, and boost happiness. It also makes older adults recall positive images. Prior studies have found that travel positively affects life satisfaction [16, 17] and mood change [18]. Some studies argued that the relationship between travel and depression could be an artifact of a baseline mental health condition, as people living with depression may have continuously low moods and feelings of helplessness or sadness that discourage them from engaging in activities that improve their mood, such as travel [19].

Having better mental conditions favor planning to trip and going on a trip itself enhances mental and cognitive health, by reducing psychological stress. Little has been documented on the reciprocal link between travel and mental well-being with age. We postulated that depression and travel have a bidirectional relationship. To identify this association and underlying pathways, we used a nationally representative population from the Korean Longitudinal Study of Ageing (KLoSA).

## Methods

### KLoSA

To collect and build statistical datasets for the purpose of implementing effective social policies for the aging society in South Korea, the KLoSA study was conducted via face-to-face interview to survey about 10,000 adults aged 45 and older living across the 15 districts of the municipal cities and provinces that comprise the country (https://survey.keis.or.kr/klosa). Participants were selected using a systematic sampling stratified by area and residential types, creating a national representative sample. The study has followed up biannually with tested individuals since 2006.

### Study population

We used 9731 participant data obtained from the 2008 to 2016 waves of the KLoSA. We excluded 1197 participants who did not respond to the depression and travel questionnaires in 2008 or who did not respond at all between 2010 and 2016. We also excluded 10 participants who responded to only one of the depression or travel questionnaires. Ultimately, we analyzed 8524 participant datapoints for this study (2010, *n* = 7847; 2012, *n* = 7111; 2014, *n* = 6690; 2016, *n* = 6252). Our study was determined to be exempted from full review by the institutional review board of the University of Seoul (exempt no. 2019-37).

### Depression and travel

To measure depression, we used the Center for Epidemiological Studies Depression Scale: 10-Items (CES-D10) of Korea version. The CES-D10 is a shorter version of the CES-D, which was developed to identify depressive symptoms related to major or clinical depression. The Korean CES-D was validated as a reliable screening tool and detailed information was provided in other studies [20]. Each item is scored with either a 0 or 1 and the total score ranges from 0 to 10 points, the sum of all item scores. Higher total scores indicate worse depressive symptoms. Using optimal cut-off point 4, sensitivity was 97% and specificity was 84% so we considered a cut-off of 4 or more as indicating depression [21]. The correlation coefficient between the CES-D and CES-D10 is high (*r* = 0.88) [22]. The Cronbach’s alpha value was 0.80 at the time of development [22]. To assess individual travel patterns, the KLoSA surveyed the number of times the respondent traveled, engaged in tourism, and went on outings in the past year.

### Statistical analysis

With regard to travel, we classified participants into the following two groups: those who had not traveled in the past year and those who had traveled at least once. Regarding depression, we classified participants into the following two groups: those with depression (i.e., ≥ 4 points on the CES-D10) and those without (i.e., < 4 points on the CES-D10). Potential confounders and travel status were compared between the people with depression and the ones without depression using the Student *t*-test and Chi-squared test.

We employed generalized estimating equation models to examine the effects of travel on the incidence of depression, excluding people who diagnosed depression in 2008. In terms of the equation with travel as an independent variable, we excluded those who never traveled in baseline year. We then used a cross-lagged panel model across three waves (2007–2008, 2011–2012, and 2015–2016) to explore the bidirectional relationship between the number of times an individual traveled and their CES-D10 score. In both models, we adjusted the following potential confounders measured in 2008: age (continuous) and sex (male or female), residential area (urban or rural), marital status (married or other), education level (elementary school or below, middle or high school, or college or above), living alone (yes or no), hypertension (yes or no), diabetes mellitus (yes or no), regular exercise (> 150 min/week, ≤ 150 min/week, or no exercise), household income (continuous: total income divided by the square root of household size), and employment status (currently active or inactive). We used Mplus version 8.0 (Muthen and Muthen 1998–2017) for the cross-lagged panel modeling and SAS version 9.4 (SAS Institute, Cary, NC) for the other analyses. We considered *P*-values less than 0.05 to be statistically significant.

## Results

In the first wave, around 15.2% had depression and only 20.4% of depressed people had gone on a trip in the previous year (Table 1). Their mean age was 63.1 years (SD = 10.5). Participants with depression tended to be older, female, and live in urban areas. Many were unmarried or divorced, while fewer were widowed. They tended to be less educated, live alone, and suffer from hypertension or diabetes. They were less likely to exercise and tended to be poorer, more economically inactive, and travel less than those without depression.

**Table 1:** General characteristics of participants

Variables	Depression status (CES-D10 point)		*P*
	No depression (< 4 points)	Depression (≥ 4 points)	
*n* (%)	7190 (84.8)	1291 (15.2)	
Age, year (SD)	62.4 (10.5)	70.1 (10.8)	< 0.001
Female, %	54.2	68.1	< 0.001
Residential area, urban, %	76	71.7	0.001
Married, %	81	59.3	< 0.001
Educational level, %			< 0.001
Elementary school or below	41.1	69.7	
Middle school or high school	47.1	26.3	
College or above	11.9	4	
Living alone, %	8.4	18.7	< 0.001
Hypertension, %	30.1	43.6	< 0.001
Diabetes, %	12.3	22.6	< 0.001
Regular exercise, %			< 0.001
≥ 150 min/week	30.4	17.1	
< 150 min/week	7.9	4.9	
No exercise	61.7	78	
Household income, 10^4^won (SD)	1558 (1688)	992 (2681)	< 0.001
Economic inactive, %	53.8	81.8	< 0.001
Travel, tourism, and outing, %	41.4	20.4	< 0.001

The descriptive sample was based on the first wave. 1 US dollar = 1200 Korean won

Participants who did not travel in the previous year had a 71% higher risk of depression in the subsequent year than those who had traveled [relative risk (RR) = 1.71, 95% confidence interval (CI) 1.52 to 1.94, *P* < 0.001] (Table 2). Participants with depression were more than 2.15 times more likely not to travel than those without depression (RR = 2.05, 95% CI 1.87 to 2.30, *P* < 0.001).

**Table 2:** The relative risk (95% confidence interval) of bidirectional relationship between no travel and depression using general estimating equation modeling

Independent variable	Dependent variable	Model	RR (95% CI)	*P*
No travel	Depression	1	1.94 (1.73–2.19)	< 0.001
		2	1.75 (1.55–1.97)	< 0.001
		3	1.71 (1.52–1.94)	< 0.001
Depression	No travel	1	1.86 (1.71–2.02)	< 0.001
		2	1.79 (1.64–1.96)	< 0.001
		3	2.08 (1.87–2.30)	< 0.001

Model 1: no adjust, Model 2: adjusted for age and sex, and Model 3: further adjusted for residential areas, marital status, education level, living alone, hypertension, diabetes, regular exercise, household income, and employment status

**Fig. 1:**
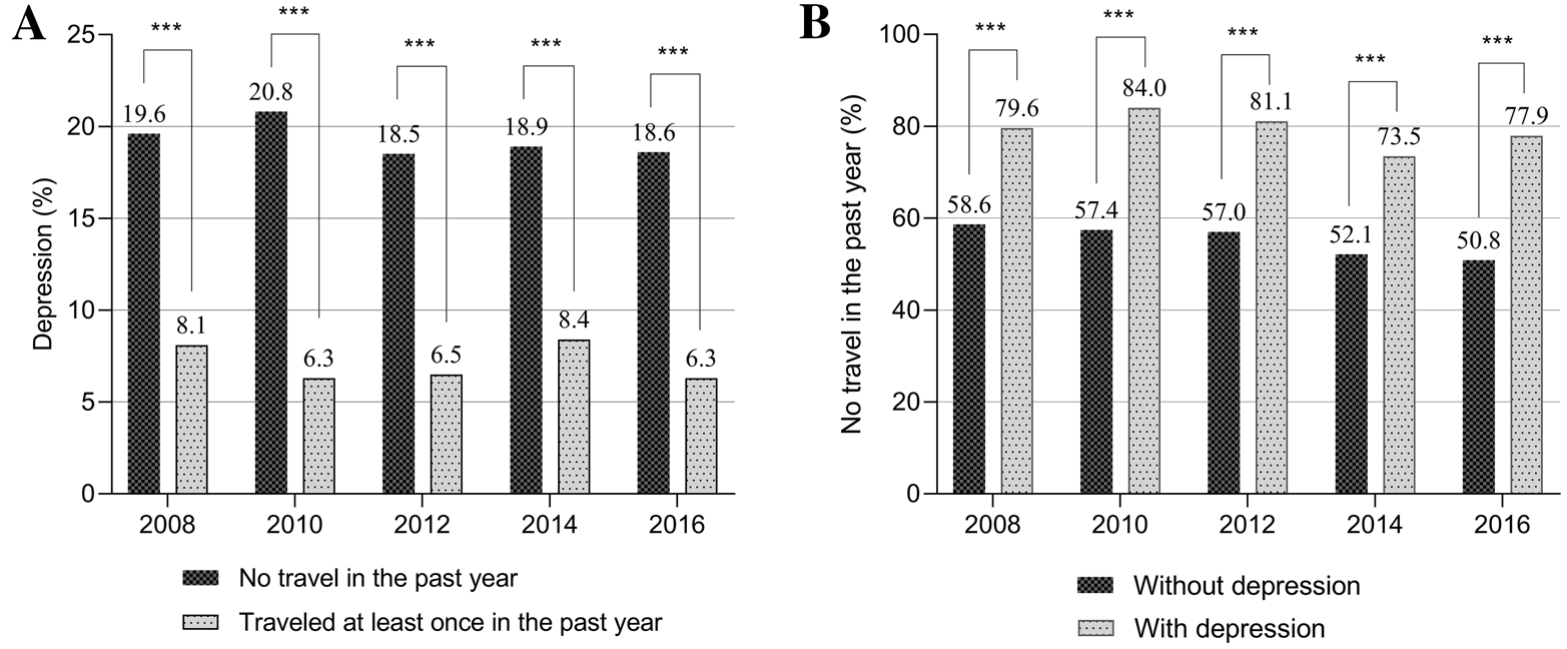
Depression and travel: (**A**) the percentage of depression according to travel experience; (**B**) the percentage of no travel according to depression status. ***P < 0.001

In the relationship between depression and travel from 2008 to 2016, the percentage of people with depression among those who never traveled in the previous year was 83.97–73.45% and the proportion of people without travel in the past year among those who were depressed was 20.75–18.46% (Fig. 1).

**Fig. 2:**
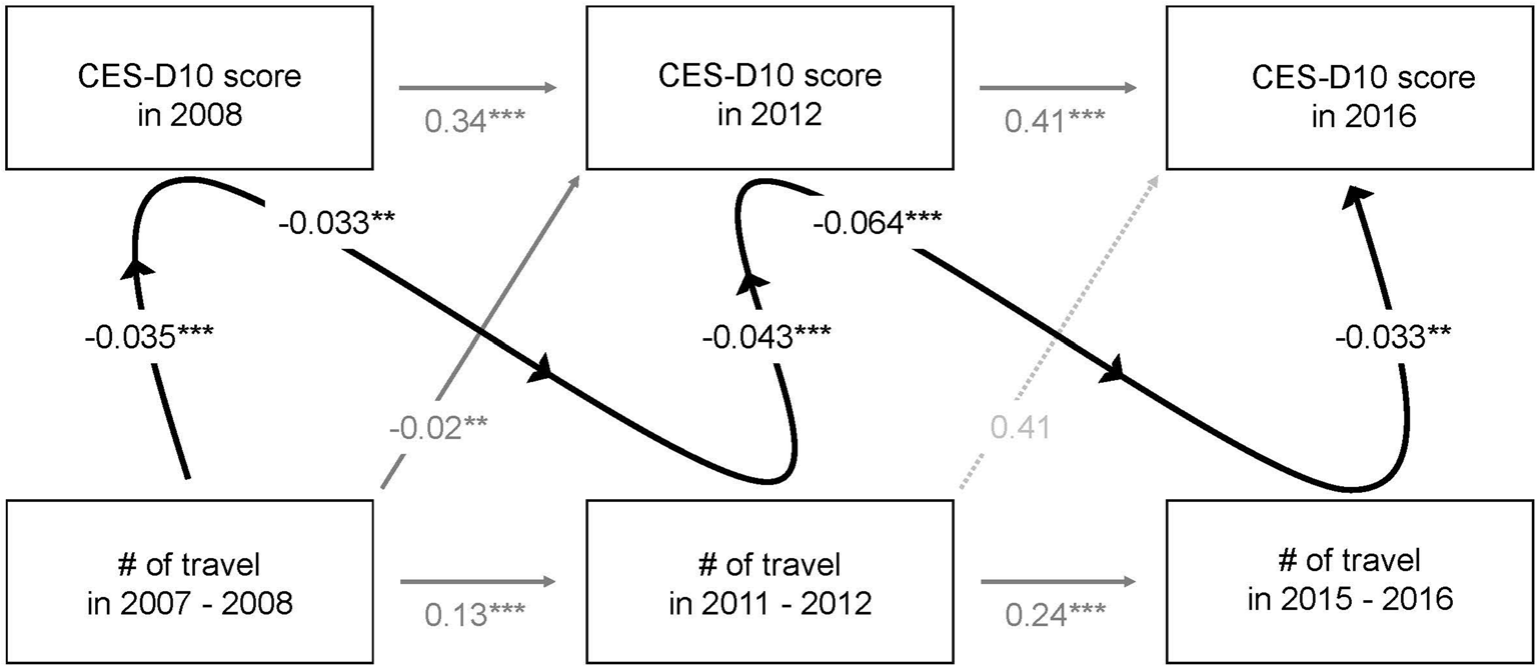
The cross-lagged panel model of relationship between the number of travel and depression score. Solid and dashed arrows indicate significant and nonsignificant relationships, respectively. The relationships of interest of the current study are highlighted by thick arrows. Numbers on arrows indicate coefficients. **P < 0.01, ***P < 0.001. Adjusted for age, sex, residential areas, marital status, education level, living alone, hypertension, diabetes, regular exercise, household income, and employment status

The cross-lagged panel model showed that participants who had traveled little between 2007 and 2008 scored significantly higher on the CES-D10 in 2008, indicating their having more depressive symptoms (*B* = − 0.035, 95% CI − 0.048 to − 0.022, *P* < 0.001) (Fig. 2). Participants with higher CES-D10 scores in 2008 tended to travel less between 2011 and 2012 (*B* = − 0.033, 95% CI − 0.053 to − 0.013, *P* = 0.007). In turn, less traveling in 2011 and 2012 increased their CES-D10 scores in 2012 (*B* = − 0.043, 95% CI − 0.061 to − 0.025, *P* < 0.001). Similar patterns were found in the 2016 wave of the survey.

We performed sensitivity analyses to examine the robustness of our results to alternative ways of depression coding criteria; results were highly consistent. We added interaction terms for the depression symptoms. We could not find statistically significant interaction effects between sex and educational attainment and cohort differences in the relationship.

## Discussion

To maintain psychological well-being over the life course is argued to be a crucial public health issue as economic burden on the government for supporting increased number of aging populations increased in many countries. Mental health issue is specifically important for older adults in Korea which is one of the fastest aging societies in the world. Our study using Korean representative sample found a bidirectional relationship between not traveling and depression. Specifically, not traveling was significantly associated with the risk of depression prospectively. Depression was also significantly associated with avoiding travel. The cross-lagged panel models analyzing each wave showed a vicious cycle between less travel and increased depressive symptoms: a reduced number of trips worsened the state of depressive symptoms, and this heightened depression resulted in fewer trips.

We found that participants who did not travel had an increased risk of depression the following year over those who had traveled. Though no previous epidemiologic study has investigated whether or how traveling prevents depression, there are several potential mechanisms that likely prompt this phenomenon. First, being distracted from routine life patterns can refresh oneself, which can be effective to reduce symptoms of depression [10, 11]. Travel contributes to recalling happy memories while positively affecting emotional condition [11]. Second, being separated from DLEs that cause depression [23, 24] may also relieve depressive symptoms. Third, meeting new people and having unique social interactions while developing peripheral social ties may play an important role in preventing social isolation among older adults [15, 25]. A study using Korean longitudinal data documented that having informal social engagement contributed to declining the symptoms of dementia and depression [26]. Given that many Korean older adults are likely to go on a trip as an activity of informal social engagement (friendship/alumni gatherings), it is plausible that the process to plan a travel positively affects mental health outcome by reducing loneliness and social isolation.

Our study provided evidence that depression can discourage traveling. The results were consistent with prior studies; major depressive disorder lowered the level of physical activity and increased the level of sedentary behavior [27]. In addition, depression may act as a barrier to engaging in healthy behaviors [28, 29] and social gatherings [19]. Moreover, depression can cause people to be pessimistic, fearful, and anxious [30], possibly making them reluctant to travel outside of their comfort zone, with others or alone.

Our research has several strengths. First, to our knowledge, this is the first study to examine the relationship between depression and traveling. Second, the nature of KLoSA, a prospective longitudinal study, allows for inferences of causality from our findings. Third, we used nationally representative sample data, and thus can extrapolate our findings to the total population of older adults in Korea.

Despite these strengths, there are still some limitations. First, this research is based on self-reported data. Therefore, recall bias may be a factor. For example, the number of trips taken in the past year could have been reported inaccurately. Second, because we only studied Koreans, more research on other populations is needed to generalize our findings. Third, KLoSA did not offer detailed information on travel (e.g., group tour/traveling alone, business/private travel, long/short distance), and thus further analysis was not possible. Finally, we could not find meaningful difference across cohorts in this model. Given that we only focused on adults aged over 45 years, further study with panel data having a wider age range should be needed to test cohort changes over time.

## Conclusions

The current study addressed that people living with depression were less likely to travel, and individuals living without travel were more likely to experience depression. The role of leisure time and informal social activities in mental well-being may become more significant with age. Our findings imply that more attention should be paid to older adults who do not travel with regard to depressive symptoms, and that travel may be beneficial for people who suffer from or are at risk of depression. Future studies should confirm our results in other populations and investigate the effects of diverse travel types on different depressive symptoms.

## Data Availability

The datasets supporting our study findings are available in the Korea Employment Information Service (https://survey.keis.or.kr/klosa).

## Abbreviations

CES-D10: Center for Epidemiological Studies Depression Scale: 10-items
CI: Confidence interval
DLE: Daily living environment
KLoSA: Korean Longitudinal Study of Ageing
RR: Relative risk
